# Emotional Availability Modulates Electrophysiological Correlates of Executive Functions in Preschool Children

**DOI:** 10.3389/fnhum.2016.00299

**Published:** 2016-06-23

**Authors:** Henriette Schneider-Hassloff, Annabel Zwönitzer, Anne K. Künster, Carmen Mayer, Ute Ziegenhain, Markus Kiefer

**Affiliations:** ^1^Department of Child and Adolescent Psychiatry and Psychotherapy, University Hospital UlmUlm, Germany; ^2^Department of Psychiatry and Psychotherapy, Ulm UniversityUlm, Germany

**Keywords:** executive function, EEG, Go/Nogo, child development, emotional availability, autonomy support, parenting, parent–child interaction

## Abstract

Executive functions (EFs) – a set of cognitive control abilities – mediate resilience to stress and are associated with academic achievement and health throughout life. They are crucially linked to prefrontal cortex function as well as to other cortical and subcortical brain functions, which are maturing throughout childhood at different rates. Recent behavioral research suggested that children’s EFs were related to parenting quality and child attachment security, but the neural correlates of these associations are unknown. With this study we tested in 4- to 6-year-old healthy children (*N* = 27) how emotional availability (EA) of the mother-child-interaction was associated with behavioral and electrophysiological correlates of response inhibition (a core EF) in a Go/Nogo task, using event-related potential recordings (ERPs), and with behavioral performance in a Delay of Gratification (DoG) and a Head-Toes-Knees-Shoulders task (HTKS). Our data showed that the Go/Nogo task modulated children’s ERP components resembling adult electrophysiological indices of response inhibition - the N2 and P3/LPC ERPs-, but the children’s N2 and P3/LPC ERPs showed longer latencies. Higher maternal autonomy-fostering behavior and greater child responsiveness were significantly associated with smaller children’s N2 Go/Nogo effects at fronto-central and parietal sites and with greater Go/Nogo effects in the N2 time window at occipital sites, over and above children’s age and intelligence. Additionally, greater maternal sensitivity and a higher dyadic EA quality of the mother-child-interaction went along with greater occipital Go/Nogo effects in the N2 time window, but this effect clearly diminished when we controlled for children’s age and intelligence. Higher maternal autonomy-support was also positively associated with better HTKS performance, and higher dyadic EA quality went along with higher HTKS and DoG scores. However, no significant associations were found between EA variables and the behavioral response inhibition measures of the Go/Nogo task. Our results suggest that parenting qualities modulate the functionality of neural circuits involved in response inhibition, an important component of EFs. This finding, thus, indicates that parent–child interactions shape the neurocognitive development underlying EFs.

## Introduction

Parenting and the quality of parent–child relationship are crucial for children’s emotional development and the establishment of attachment patterns (Bowlby, 1969; Ainsworth and Bell, 1970). However, there is growing evidence that the quality of parent–child relationship also shapes children’s EFs skills (e.g., Belsky et al., 2007; Bernier et al., 2010, 2012; Blair et al., 2011; Dilworth-Bart, 2012; Towe-Goodman et al., 2014; Bindman et al., 2015). The term EFs refers to higher-order cognitive skills, that allow for flexible, adaptive and goal-oriented behavior (e.g., Norman and Shallice, 1986). EFs have considerable impact on personal well-being over the life course and on societal prosperity: They are considered as “vital for human autonomy” and are associated with social competence, academic achievements, socioeconomic status, stress coping, health-related behaviors (e.g., substance abuse), physical and mental health, and criminality (e.g., Mischel et al., 1989; Morgan and Lilienfeld, 2000; Royall et al., 2002; Blair and Razza, 2007; Best et al., 2011; Moffitt et al., 2011; McClelland et al., 2014).

According to a predominant theory of EF, proposed by Miyake et al. (2000), the EF construct comprises three separable, but interrelated components – i.e., working memory updating, inhibition, and shifting. The interrelations between the dissociable components point to a “common mechanism across different EFs” (Miyake et al., 2000). There is not yet a consensus about the quality of this common EF mechanism. Several researchers have suggested that an attention system constitutes the core of EFs, e.g., as proposed in the framework of the supervisory attentional system (Norman and Shallice, 1986), the theory of the executive attention network (Rueda et al., 2004; Posner and Rothbart, 2007) and in the concept of the controlled attention system (Engle et al., 1999).

Zelazo and Müller (2002) put forward to additionally distinguish between ‘cool’ and ‘hot’ EF (Zelazo and Carlson, 2012). ‘Cool’ EFs refer to abstract, affectively neutral contexts, while ‘hot’ EFs involve the top-down control of motivation and affect. Different brain areas are assumed to subserve ‘cool’ and ‘hot’ EFs: ‘Cool’ EFs possibly recruit the lateral prefrontal cortex and ‘hot’ EFs orbitofrontal and other medial prefrontal cortex areas (Zelazo and Carlson, 2012).

Executive functions were originally conceptualized as exclusively frontal cortex function. However, lesion and neuroimaging studies revealed that the frontal cortex is neither specific nor sufficient for executive functioning and that EFs involve an extended network encompassing frontal, parietal, temporal, and occipital cortices as well as subcortical areas (Alvarez and Emory, 2006). The interpretation of these findings is nevertheless complicated by the fact that EFs control the production of activities and thereby interact with and depend on other processes such as for instance perception or memory (Royall et al., 2002; Alvarez and Emory, 2006). This issue leads to the so-called task impurity problem in the measurement of EFs: Tasks that are designed to measure EF proficiency not only involve the target EF, but other cognitive processes as well (Miyake et al., 2000).

The prefrontal cortex matures at a slower rate than most other brain areas, with highest plasticity until age 7, and its maturation protracts into young adulthood (Huttenlocher and Dabholkar, 1997; Gogtay et al., 2004). A similar developmental pattern has been observed for EFs. EFs develop over childhood and adolescence into adulthood (Garon et al., 2008; Best and Miller, 2010) and show moderate stability over the life course (e.g., Mischel et al., 1989; Casey et al., 2011). It is assumed that brain maturation parallels the evolution of children’s EF abilities (e.g., Garon et al., 2008; Best and Miller, 2010). A comparative study with human infants and primates provided evidence that brain maturation of prefrontal cortices likely underlies the developmental improvements in EFs during the first year of life (Diamond and Goldman-Rakic, 1989). Infancy and early childhood are considered pivotal for the EF development (Garon et al., 2008; Best and Miller, 2010). Rudiments of the EF components inhibition, working memory updating and shifting, as well as of voluntary attention are present as from the first year of life (Garon et al., 2008). Rapid growth of EF occurs in preschool and early school years, and children become able to deal with more complex EF tasks (Garon et al., 2008; Best and Miller, 2010). From 3 to 5 years of age all three EF components (i.e., inhibition, working memory updating, shifting) improve significantly (e.g., Carlson, 2005; Garon et al., 2008). The observed EF improvements could be either attributed to quantitative changes in each EF component and/or to changes in the underlying common factor, as was put forward by Garon et al. (2008), for instance.

Mammalian brain maturation is not solely genetically determined, but relies upon appropriate, species-typical stimulation from the environment (experience-expectant maturation) and it is shaped by environmental stimuli, that may be unique to an individual (experience-dependent maturation; Greenough et al., 1987). Animal studies revealed that variations in the environment have a strong impact upon animal’s brain structure, with varying effects at different ages (e.g., Kolb et al., 1998). However, not only the physical environment, but also the emotional quality of caregivers’ interactions with the child are important for brain and cognitive-emotional development (Harlow and Harlow, 1962). With regard to humans, children’s brain structure and functioning is affected by adverse rearing conditions (e.g., Sheridan et al., 2012), and ordinary variations in maternal sensitivity and intrusiveness are associated with differences in children’s frontal brain activity (Hane and Fox, 2006). Developmental research, in particular attachment theory, highlighted the importance of the caregiver–child relationship on children’s cognitive, social, and emotional growth (Harrist and Waugh, 2002; Sroufe, 2005; Waller et al., 2015). It has been assumed that caregivers initially serve as external regulators of the infant and facilitate the progression from external to internal regulation, depending on parental sensitivity to the child (e.g., Kopp, 1982). Parental sensitivity, defined as prompt, contingent, and appropriate response to infant’s behavior, is closely linked to child attachment security (e.g., Bowlby, 1969; Blehar et al., 1977). A secure attachment provides the child with a secure base for competent exploration of the environment (Ainsworth and Bell, 1970). The opportunity for exploration likely allows the child to develop and practice self-controlled actions (see e.g., Bernier et al., 2012). Child attachment security is not only determined by parental sensitivity, but also by parental autonomy-support (Whipple et al., 2011) and mind-mindedness (Meins et al., 2001). Mind-mindedness describes the parental tendency to perceive the child as individual with own mental states, and to appropriately comment on the child’s mental states (Meins et al., 2001). Autonomy-support is defined as the parental tendency to recognize and value children’s needs, to support their choices and their independent problem-solving. It involves scaffolding behavior that offers age-appropriate problem-solving strategies to the child. Carlson (2003) proposed that maternal sensitivity, mind-mindedness and autonomy-support might be crucial facilitators of children’s EF development.

As outlined above, individual differences in EFs may be accounted for by genetic and/or (social) environmental factors. Twin and genetic association studies suggest that EFs are under considerable genetic control (Friedman et al., 2009; Barnes et al., 2011). Similar results were obtained for the attention network (Fan et al., 2001; Barnes et al., 2011).

However, the social environment proved to be relevant for EF proficiency as well, as revealed in several cross-sectional or longitudinal studies that investigated home environment characteristics and different aspects of parenting in their relation to children’s EF:

Lower socioeconomic status (operationalized as maternal education and household income) was significantly associated with worse performance in EF tasks in children (at ages 4–5; Dilworth-Bart, 2012). Parental education (but not occupation or income-to-needs ratio) accounted for about 12% of variance in EF performance in children (mean age 5 years), with lower parental education predicting lower EF task scores (Noble et al., 2005).

Maternal sensitivity at 1 or 2 year(s) predicted EF capacity in children at ages 18 months to 3 years (Bernier et al., 2010, 2012; Towe-Goodman et al., 2014). Similarly, greater maternal sensitivity at 54 months and at 6 years was associated with better attentional control at ages 6 and 9 (Belsky et al., 2007), and greater maternal responsiveness at 22 months (albeit not at 9 nor 14 months) was related to higher effortful control (a construct overlapping with EFs) in children aged 22 and 33 months (Kochanska et al., 2000). Father’s sensitive caregiving (Bernier et al., 2012; Towe-Goodman et al., 2014), maternal scaffolding (Bibok et al., 2009; Hughes and Ensor, 2009; Hammond et al., 2012) and maternal mind-mindedness (Bernier et al., 2010, 2012) were significantly associated with children’s EF outcomes. The latter also predicted EF improvements between 18 and 26 months better than other parenting variables (Bernier et al., 2010). In studies that investigated parenting multidimensionally, attachment security, and maternal autonomy-support emerged to be most predictive for EF performance (Bernier et al., 2010, 2012; Bindman et al., 2015). Child attachment security at ages 12 and 18 months also predicted DoG proficiency in a ‘hot’ EF task in 6-year-olds (Jacobsen et al., 1997) and higher maternal responsiveness and autonomy-support across the first 3 years were significantly related to more proficient DoG at 54 months (Razza and Raymond, 2013; Bindman et al., 2015).

While the importance of attachment-related experiences for EF development has been demonstrated in several studies, little attention has been paid on whether and how these experiences are associated with EF-related neural processes in children. We hypothesized that the quality of parent–child interactions modulates neural processes that underlie children’s EF performance. In the present study, we investigated how the quality of caregiver–child interactions is associated with behavioral and electrophysiological measures of EFs.

We chose EA as construct to assess the quality of the caregiver–child interactions. EA “refers to a dyad’s capacity for emotional connection and the extent to which the connection is genuinely affectively positive and healthy and the extent to which the dyad can accommodate and downregulate negative affect” (Biringen and Easterbrooks, 2012b). The EA construct is considered to contain attachment as a component. EA focuses more on positive emotions compared to attachment theory and can be assessed across a broader range of situations (Biringen and Easterbrooks, 2012b). Several studies demonstrated that key aspects of the caregiver–child relationship can be measured by the EA scales in a reliable and valid manner (see e.g., Biringen and Easterbrooks, 2012b; Bornstein et al., 2012).

For the assessment of EFs, we chose behavioral tasks that are well established in developmental research: (a) The HTKS, that involves all three dimensions of EF and refers to the concept of ‘cool’ EF, (b) the DoG, that implicates impulse control and refers to ‘hot’ EF, and (c) the Go/Nogo task, that targets (behavioral) response inhibition and has been intensively studied in electrophysiological research. During the Go/Nogo task event-related potentials (ERPs) were recorded to reveal the electrophysiological correlates of EF.

The Go/Nogo task allows for the minimization of other cognitive and behavioral processes, thereby reducing the task-impurity problem. The Go/Nogo task involves two conditions: In the ‘go’ condition participants have to respond to a given target stimulus. In the ‘nogo’ condition subjects have to refrain from a response to a given stimulus. In electrophysiological studies in adult or adolescent participants the effect of response inhibition (Go/Nogo effect) was shown to be associated with two event-related potentials, the N2 and the P3 component (Kopp et al., 1996; Kiefer et al., 1998; Stroth et al., 2009). The N2 component is a negative ERP that peaks over fronto-central sites between 150 and 350 ms after stimulus presentation, with a larger amplitude in the ‘nogo’ condition compared to the ‘go’ condition. This N2 Go/Nogo effect is assumed to be associated with higher-order inhibitory processes (Eimer, 1993; Kiefer et al., 1998; Ruchsow et al., 2008) and/or conflict monitoring (Nieuwenhuis et al., 2003; Donkers and van Boxtel, 2004). Source analyses suggested that the ‘nogo’ N2 is generated in bilateral inferior frontal cortices (Kiefer et al., 1998). The P3 component, alternatively labeled as late positive complex (LPC), is a positive ERP that usually peaks between 300 and 600 ms after stimulus presentation. The ‘go’ P3 shows its maximum over centro-parietal sites, while the ‘nogo’ P3 is enlarged over fronto-central sites. According to source analysis the P3 Go/Nogo effect likely originates from anterior cingulate and motor cortex activity (Kiefer et al., 1998). The P3 is assumed to reflect attentional resource allocation engaged for the evaluation of stimuli, and to be related to working memory processes involving context updating and subsequent memory storage (Polich, 2007). Comparably to the N2, the P3 Go/Nogo effect is regarded as signature of response inhibition (Kiefer et al., 1998; Smith et al., 2008). On the basis of neuroimaging data a model for behavioral response inhibition was posited by Aron (2007): Successful inhibition possibly activates a fronto-subthalamic circuit (i.e., inferior frontal cortex and subthalamic nucleus) and subsequently leads to the inhibition of the primary motor cortex via basal ganglia/thalamo-cortical pathways.

Developmental neuroimaging and electrophysiological studies of response inhibition suggested immature prefrontal cortex functioning as well as different task strategies in early and middle childhood compared to adults (Bunge et al., 2002; Davis et al., 2003; Jonkman et al., 2003; Ciesielski et al., 2004; Jonkman, 2006). With regard to behavioral measures children usually preformed less accurate (Casey et al., 1997; Bunge et al., 2002; Davis et al., 2003; Jonkman et al., 2003; Ciesielski et al., 2004) and with slower reaction times (Bunge et al., 2002; Davis et al., 2003; Ciesielski et al., 2004; Lamm et al., 2006) than adults in response inhibition tasks. Electrophysiological research additionally revealed decreases in latencies and amplitude of the N2 (Davis et al., 2003; Jonkman et al., 2003; Jonkman, 2006; Lamm et al., 2006) and P3 (Davis et al., 2003) ERP components as a function of children’s age. Decreases in ‘nogo’ N2 amplitude were associated with better EF performance over and above effects of age (Lamm et al., 2006). Absent ‘nogo’ P3 in children (ages 6–7, respectively, 9–10) was reported by Jonkman et al. (2003) and Jonkman (2006) and different P3 patterns in 6-year-old children compared to adults were observed by Davis et al. (2003). These developmental changes in the N2 and P3 components might reflect ongoing maturation of the prefrontal cortex.

Earlier ERP components than the N2 or P3, such as the N1 ERP component, are usually disregarded for the evaluation of response inhibition in Go/Nogo tasks. However, an enlargement of the N1 in the ‘nogo’ relative to the ‘go’ condition was observed by Filipović et al. (2000) and by Lavric et al. (2004). The N1 component is a negative ERP that peaks between 125 and 175 ms over parieto-occipital sites for visual stimuli (Harter et al., 1982; Luck et al., 1990). Focused attention leads to an enlargement of the N1 (e.g., Luck et al., 1990). It has been proposed that the enlarged N1 component refers to an early visual selection process and represents the orienting and/or recruiting of an attention system to a task-relevant location (Luck et al., 1990). It was assumed that the N1 ‘nogo’ effect might trigger the later inhibitory process (Lavric et al., 2004) and might be more specifically related to the ‘nogo’ decision than later components (Filipović et al., 2000).

As outlined above, we aimed at investigating whether and how parenting is associated with EF-related neural processes in children, using ERP recordings. To our knowledge, this issue has so far not been addressed by other studies. We hypothesized that the quality of parent–child interactions modulates neural processes that underlie children’s EF performance. In the present study, we investigated how the quality of caregiver–child interactions is associated with behavioral and electrophysiological measures of EFs. The interaction quality was assessed with the construct of EA. For the assessment of children’s EF ability, the HTKS, DoG and Go/Nogo tasks were chosen. Children’s ERPs were recorded during Go/Nogo performance. Children were 4- to 6-year-old. We hypothesized that the Go/Nogo effects on the N1, N2, and P3 ERP components are related to the behavioral EF task performance indicating that electrophysiological measures are indicative of response inhibition performance.

We also predicted that higher EA scores are associated with (a) better behavioral EF task performance and with (b) Go/Nogo effects of the ERP components N1, N2, and P3 that are indicative of more efficient EF skills.

## Materials and Methods

### Participants

Data from 27 parent–child dyads were used for the analysis. Mean age of the children (*n* = 27, four sibling pairs) was 58.7 months (*SD* = 6.6, range 48.1–72.8; 48.2% girls). Mothers (*n* = 23) were between 29.4 and 48.6 years old (*M* = 39.0, *SD* = 4.0), and had either a master’s degree (63.0%) or an otherwise completed professional education (37.0%). All mothers were married or living with the child’s father.

Families were recruited in kindergartens of Ulm and Neu-Ulm (Germany). Inclusion criteria were children’s age (48–73 months), mastery of the German language and absence of any known psychiatric or neurological disease or severe developmental delay according to parent report. A total of 36 children was recruited. For eight children EEG data were not available due to technical problems or due to discontinuation of the EEG experiment. These data sets were therefore excluded. One child was excluded because of low performance in the intelligence task (i.e., below two standard deviations according to normative, age-adjusted data (Bulheller and Häcker, 2002)). Mothers gave written informed consent and the study protocol was approved by the local ethics committee according to the declaration of Helsinki.

### General Procedure

The current study involved two laboratory visits for the mother–child dyad, typically within 2 weeks. During the first visit children were administered several behavioral EF tasks and one intelligence test. In order to assess EA, mother and child engaged in free play and their interaction was videotaped. A questionnaire battery was delivered to the mother including reports on family demographics and child’s behavior. During the second visit children participated in a Go/Nogo task, which measures EFs, and during which task-related electroencephalographic activity was recorded.

#### Assessment of Emotional Availability

Mothers and children were instructed to play at a table with toys in a typical manner, and to tidy the table at the end of the play. The duration of this free-play interaction was at least 20 min. The mother–child interaction was videotaped and the interactions were coded for maternal and child’s behavior using the fourth edition of the EA scales (Biringen, 2008).

Emotional availability is a global measure of “dyadic or relational capacity for mutual emotional awareness, perception, experience, and expression” (Biringen et al., 2005; Biringen and Easterbrooks, 2012b). The EA measure has been empirically validated for parents of children aged 0–14 years (Biringen and Easterbrooks, 2012b). Its operationalization comprises four adult components (sensitivity, structuring, nonintrusiveness, nonhostility), two child components (responsiveness, involvement) and the EA CS. Adult sensitivity assesses warmth and attunement to emotional cues. Adult structuring refers to guidance of child’s play and to setting limits, and it also involves autonomy-fostering behaviors. Encouragement of age-appropriate autonomy by the absence of interference, overprotection or withdrawal is operationalized as nonintrusiveness. Adult nonhostility describes the emotional range from absent hostility to covert or open hostile behavior. Child responsiveness is characterized as child’s behavioral and emotional responsiveness comprising compliance. Lower scale points either indicate over- or under-responsiveness of the child. Child involvement describes child’s initiative to engage the adult in the interaction. Finally, the EA CS is a global measure of the adult–child relationship quality, with an emphasis on adult sensitivity and child responsiveness (Biringen and Easterbrooks, 2012a,b).

The adult and child components are rated on a 7-point Likert-type scale (*D*-scores) and on a 29-point rating scale (*T*-scores), the latter are a sum of seven subscales. High scores represent the optimal level of EA and low scores indicate complicated EA. The EA CS ranges from 1 to 100 points; high EA CS scores (>80) indicate a dyadic EA. *D*-scores and *T*-scores were highly correlated (ρ ≥ 0.88) and *D*-scores yielded results that were similar to those of the *T*-scores (data not shown).

However, as *T*-scores are assumed to “more fully capture the variability among cases” (Biringen and Easterbrooks, 2012a), further data analyses were based on *T*-scores. We additionally summed up the *T*-scores of the four adult and the two child EA scales to the variable “EA sum.”

Emotional availability coding uses verbal and non-verbal indicators (like physical, facial, and vocal signals as well as displays of positive and negative emotions), with an emphasis on non-verbal cues. Coding was done by one research assistant who had obtained inter-rater reliability with Biringen. Five dyads were coded by a second trained assistant and inter-rater reliability was very good for five of the EA scales (adult structuring, adult nonintrusiveness, adult nonhostility, child responsiveness, child involvement; ICC > 0.89, *p* < 0.01), acceptable for adult sensitivity (ICC = 0.67, *p* = 0.052), but rather low for EA CS (ICC = 0.55, *p* = 0.10).

Emotional availability scores of one child were missing due to technical problems with the video equipment. We expected that EA is not stochastically independent for siblings; therefore we excluded one child of each sibling pair at random, resulting in a sample size of 22 dyads.

#### Behavioral Executive Functions Tasks

##### Head–toes–knees–shoulders task (HTKS)

The HTKS task is a measure of cognitively mediated behavioral self-regulation that involves attention, cognitive flexibility, working memory, and inhibitory control, i.e., components of EFs (Cameron Ponitz et al., 2008; McClelland et al., 2014). The task is appropriate for children aged 4–8 years. The HTKS comprises 30 test items, and scores range from 0 to 60. Higher scores imply higher levels of EFs. The HTKS is divided into three sections with 10 items each, and involves up to four paired behavioral rules (“touch your head/toes/knees/shoulders”): In the first section, children have to respond in a non-automatic way (i.e., touching their toes when told to touch their head, and vice versa), in the second section, a paired behavioral rule is added (i.e., touching their knees when told to touch their shoulders, and vice versa), and in the third section, the rules are changed by switching the pairings (i.e., toes go with shoulders and head goes with knees). The task was shown to be reliable and valid, to have good test–retest stability and to significantly predict academic achievements (McClelland and Cameron, 2012; McClelland et al., 2014). In this study, we administered the validated German version (von Suchodoletz et al., 2014).

##### Delay of gratification task (DoG)

The DoG task measures the child’s ability to “[voluntarily postpone] immediately available gratification in order to attain delayed but more valued outcomes” (Mischel et al., 1989). The task was shown to be predictive for social and cognitive competence in later life (Mischel et al., 1989). The DoG is assumed to involve cognitive control of motivation and affect (Hongwanishkul et al., 2005).

In this study, children were first asked to choose their preferred candy (Smarties, lollipop, jelly babies), which was subsequently placed in front of them. The experimenter then explained to the children that their mother and the experimenter had to leave the child for a while and that the candy was meant for the child as a reward for waiting. Children were also told that they would receive a further candy portion if they waited until the return without leaving their chair, eating the candy or ringing the bell. The child was informed that it can shorten the waiting period by ringing the bell, but that then it would only receive one instead of two candy portions. Scores were the number of seconds waited (Mischel et al., 1989). Maximum duration of the task was 15 min.

#### Control Variables

##### Colored progressive matrices task (CPM)

Child’s general intelligence was assessed with a non-verbal task, the Colored Progressive Matrices task (Raven et al., 1998; Bulheller and Häcker, 2002). The task contains 36 items, and the maximum score is 36. The percentile rank was computed for each child by use of normative, age-adjusted data (Bulheller and Häcker, 2002).

*Children*’*s age* and *maternal education* were assessed in maternal reports on sociodemographic variables.

##### Strengths and difficulties questionnaire (SDQ)

The Strengths and Difficulties Questionnaire Version P4-16 (Goodman et al., 2000; Goodman, 2001), a brief behavioral screening instrument for children aged 4–16 years, was administered to the mothers. The SDQ measures psychopathology and prosocial behavior. The questionnaire contains 25 three-point items, that are divided between 5 subscales: “emotional symptoms,” “conduct problems,” “hyperactivity/inattention,” “peer relationship problems,” and “prosocial behavior.” The first four subscales are summed up to generate a total difficulties score^1^ Children’s behavior is classified according to the sum scores (of the subscales resp. the total difficulties score) into “normal,” “borderline,” and “abnormal” behavior. In a general population 80% of the children show “normal,” 10% “borderline,” and 10% “abnormal” behavior (Goodman et al., 2000).

#### EEG Experiment

##### Go/Nogo task

The Go/Nogo task requires inhibition of a prepared response, thereby implicating inhibitory control processes. In the ‘go’ condition the participant has to respond to a given target stimulus. In the ‘nogo’ condition the subject has to withhold a response to a given stimulus. The ‘go’ stimulus was a picture of a circular wooden board. The ‘nogo’ stimulus was a picture of a cookie, i.e., a rewarding cue, which should induce approach tendencies and render the suppression of the response more difficult (see Casey et al., 2011). ‘Go’ and ‘nogo’ stimuli were selected such that shape and color of the stimuli were highly similar in order to keep visual processing of the stimuli comparable. The stimulus size was 16 cm × 11 cm (410 × 314 pixel) for ‘go’ and ‘nogo’ stimuli. We presented the ‘go’ stimulus with a higher frequency than the ‘nogo’ stimulus (80 vs. 40 trials per condition), so that participants were used to prepare and execute a response in the majority of trials, which had to be withhold in the rare ‘nogo’ condition. Stimulus duration of both ‘go’ and ‘nogo’ stimuli was 750 ms, interstimulus interval varied between 1600 and 2000 ms (*M* = 1800 ms). Children were instructed to press a key as fast and as accurately as possible only when the wooden board (‘go’ stimulus) was presented, but to withhold a response upon the cookie (‘nogo’ stimulus).

As behavioral data, we recorded the response latency in the ‘go’ trials and the response accuracy for both ‘go’ and ‘nogo’ trials. Response accuracy is reported in terms of relative frequency of correct responses in the ‘go’ trials (hits) and relative frequency of incorrect responses in the ‘nogo’ trials (false alarms). Additionally, the sensitivity measure *d′*, which is “defined as the separation, in standard deviation units, between a pair of hypothesized normal density functions representing the internally observed effects of signal plus noise, and noise alone” (Nevin, 1969), was calculated by subtracting z(false alarms) from z(hits), with z as “the inverse of the normal distribution function” (see e.g., Macmillan and Creelman, 2005). The measure *d′* hence captures performance independent of response biases according to signal detection theory (Green and Swets, 1966).

Additionally, a sum score, *EF sum*, of z-standardized HTKS, DoG and Go/Nogo *d′* scores was computed (for data reduction purposes). A factor analysis of the EF task scores was not appropriate due to the small sample size.

##### EEG recording and ERP extraction

Children were seated in a height-adjustable chair in a sound-attenuating, electrically shielded booth. EEG was continuously recorded using 39 channels mounted in an elastic cap (Easy Cap, Herrsching, Germany). Sintered Ag/AgCl-electrodes were positioned according to the extended 10–20 system. All electrodes were referenced to an electrode at the left earlobe. Eye movements were registered by vertical and horizontal electro-oculograms. Electrode impedances were kept below 5 kΩ. Electrical signals were continuously recorded with BrainAmp amplifiers (BrainProducts GmbH, Gilching, Germany) with a bandwidth of 0.01–100 Hz and a 50 Hz notch filter. The data were digitized at a sampling rate of 500 Hz. After data acquisition a Butterworth Zero Phase Filter (low cutoff: 0.20 Hz, 12 dB/oct; high cutoff: 30 Hz, 24 dB/oct) was applied to the data. EEG data were analyzed using BrainVision Analyzer 2.0 (BrainProducts GmbH, Gilching, Germany).

Continuous EEG was segmented from 150 before to 1000 ms after stimulus onset. Ocular artifacts were removed using a regression method proposed by Gratton et al. (1983) and segments were baseline corrected to an interval of 150–0 ms before stimulus onset. Artifacts were automatically rejected by use of following criteria: minimal allowed amplitude of -100 μV, maximal allowed amplitude of 100 μV and maximal allowed absolute difference of 180 μV. Afterward, data were manually checked whether all artifacts had been removed. Stimulus-locked event-related potentials (ERPs) were extracted by averaging artifact-free EEG segments for ‘go’- and ‘nogo’-trials separately. EEG epochs with delayed or erroneous responses were excluded from ERP analysis.

#### Statistical Analyses

##### ERP components

ERP components of interest were the N1, N2, and P3/LPC components. For statistical analyses, electrodes were selected in clusters over occipital, parietal, and fronto-central scalp regions, including lateral as well as midline electrodes in separate clusters, at which these components were typically largest (Coles and Rugg, 1995). Potentials were averaged across electrode sites within left and right hemisphere clusters, respectively: Left (O1) and right occipital (O2), left (cluster Par-left: P3, P7) and right parietal (cluster Par-right: P4, P8), left (cluster Fcentral-left: FC3, C3, CP3) and right fronto-central (cluster Fcentral-right: FC4, C4, CP4) as well as midline electrodes including occipital (Oz), parietal (Pz), and fronto-central (Cz) electrodes.

We identified an occipital N1 ERP component, a fronto-central N2 component and a parietal P3/LPC component. We labeled the ERP components according to their order of appearance in time because ERP latencies vary with age (see e.g., Davis et al., 2003; Jonkman et al., 2003; Itier and Taylor, 2004; Jonkman, 2006; Lamm et al., 2006).

For statistical analyses, mean amplitudes within time windows centered at the corresponding peaks of these components were computed as dependent ERP variables. The N1 time window was defined as time period from 184 to 284 ms after stimulus presentation, the N2 time window lasted from 318 to 518 ms and the P3/LPC time window from 600 to 800 ms. ERP amplitudes in the N1, N2, and partially P3/LPC time window were found to be polarity inverted at fronto-central electrode sites compared with occipital and parietal sites, similarly to previous studies (Kiefer et al., 1998; Kaiser et al., 2003).

Mean potentials at all electrode sites (i.e., occipital, parietal, fronto-central, and midline electrodes) were analyzed in repeated measures analyses of variance (ANOVAs) for each ERP time window separately, in order to reveal significant differences between ‘go’ and ‘nogo’ condition for the selected electrodes/electrode clusters. The ANOVAs included two within-subject factors for the occipital and midline electrodes: Condition (‘go,’ ‘nogo’) and hemisphere/electrode sites (occipital: O1, O2; midline: Oz, Pz, Cz). For the parietal and fronto-central clusters three within-subject factors were included: Condition (‘go,’ ‘nogo’), hemisphere (left, right) and electrode sites (parietal: P3/P4, P7/P8; fronto-central: FC3/FC4, C3/C4, CP3/CP4). We only report the main effects of condition and the interaction effects of condition and hemisphere, in order to reduce the complexity of the result section. In case of significant interactions, follow-up analyses were conducted using *post hoc t*-tests.

For correlational analyses ERP Go/Nogo effects were computed by subtracting potentials of individual ‘nogo’ ERPs from those of ‘go’ ERPs.

##### Behavioral data

Statistical analyses of the behavioral data were performed using R 3.2.1^2^ Packages in use were “car” (Fox and Weisberg, 2011), “ez” (Lawrence, 2013), and “psych” (Revelle, 2015). Repeated measures analyses of variance with three within-subject factors were carried out using STATISTICA for Windows, Version 12^3^

Associations between Go/Nogo effects, behavioral measures of EFs and measures of EA were assessed in correlational analyses. Taking into account the small sample size, Spearman’s rho correlations were computed. In addition, partial correlations were performed to control for children’s age and children’s intelligence (CPM scores): Multiple regressions were computed with children’s age and children’s CPM scores as predictors, and for the residuals the Spearman’s rho correlations were computed. In the multiple regressions the variance inflation factor (i.e., a multicollinearity measure) was 1.48 and therefore did not exceed critical values [as commonly recommended (O’Brien, 2007)].

Alpha level of statistical significance was set at *p* < 0.05 for all analyses. Because of the exploratory nature of the analyses, no correction for multiple comparisons was applied.

## Results

### Behavioral Executive Function Data

Mean values, standard deviations (SD) and observed ranges of HTKS, DoG, ‘go/nogo’ response accuracy, ‘go’ reaction time and children’s intelligence are presented in **Table 1.** Associations with children’s age are shown in the last column of **Table 1.** According to the SDQ children’s behavior was rated “normal” for 88.9% of the children and “abnormal” for 11.1%. The distribution of the SDQ ratings in our sample thereby indicated slightly lower levels of psychopathology compared to the general population (Goodman et al., 2000). Maternal education was not significantly associated with EF scores (**Supplementary Table SI1**), and this variable was excluded from the subsequent analyses. However, older children had significantly higher HTKS scores, more ‘go’ hits and a higher ‘go/nogo’ *d′*. With regard to the percentile rank of children’s intelligence no significant correlations were observed (**Supplementary Table SI1**).

**Table 1:** Descriptive information for the behavioral EF measures (*N* = 27).

Variable	*M*	*SD*	Observed range	Child’s age (ρ)
HTKS [scores]	34.48	16.47	0–57	0.60^∗∗∗^
DoG [sec]	494.07	351.41	22–900	0.32
Go/Nogo *d′*	2.97	1.44	1.06–5.96	0.61^∗∗∗^
Go Hits [rel.fr.]	0.90	0.10	0.59–1.00	0.69^∗∗∗^
Nogo False alarms [rel.fr.]	0.16	0.13	0.00–0.48	–0.30
Go RT [ms]	715.37	100.57	546.89–968.26	–0.42^∗^
EF sum [*z*-scores]	0.00	2.28	–4.16-3.88	0.64^∗∗∗^
CPM [scores]	18.26	5.47	11–33	0.54^∗∗^
CPM [pr]	69.26	26.52	24–100	0.21


*HTKS, Head–Toes–Knees–Shoulders Task; DoG, delay of gratification task; EF sum, sum of z-standardized HTKS, DoG and Go/Nogo *d′* scores; CPM, colored progressive matrices; rel.fr., relative frequency; pr, percentile rank; M, mean; SD, standard deviation; rho, Spearman’s rho correlation coefficient.*

*^∗^*p* < 0.05; ^∗∗^*p* < 0.01; ^∗∗∗^*p* < 0.001.*

Intercorrelations of the behavioral EF measures across the different tasks are shown in **Table 2.** Higher HTKS scores significantly correlated with higher DoG scores, i.e., a longer DoG waiting period, with more ‘go’ hits and a higher *d′* in the Go/Nogo task, but not with ‘nogo’ false alarms; however, when controlling for children’s age and intelligence, higher HTKS scores were only significantly associated with less ‘nogo’ false alarms. Higher DoG scores were associated with more ‘go’ hits (significantly in the zero-order correlation analysis, but insignificantly when we controlled for children’s age and intelligence). Faster ‘go’ reaction times were significantly correlated with more ‘go’ hits (in both correlations).

**Table 2:** Intercorrelations of the behavioral EF measures (*N* = 27).

Variable	HTKS	DoG	Go/Nogo *d′*	Go Hits	Nogo FAs	Go RT
HTKS	–	0.42^∗^	0.50^∗∗^	0.66^∗∗∗^	–0.37	–0.38
DoG	0.32	–	0.27	0.40^∗^	–0.13	–0.05
Go/Nogo *d′*	0.23	0.10	–	0.80^∗∗∗^	–0.82^∗∗∗^	–0.37
Go Hits	0.31	0.37	0.61^∗∗∗^	–	–0.41^∗^	–0.56^∗∗^
Nogo false alarms	–0.39^∗^	–0.04	–0.81^∗∗∗^	–0.45^∗^	–	0.11
Go RT	–0.13	0.02	–0.14	–0.43^∗^	0.05	–


*Spearman’s rho zero-order correlation coefficients are presented above the diagonal, below the diagonal the partial correlation coefficients, controlling for children’s age and intelligence, are depicted.*

*HTKS, Head–Toes–Knees–Shoulders task; DoG, delay of gratification task; FAs, false alarms; RT, reaction times; ^∗^*p* < 0.05; ^∗∗^*p* < 0.01; ^∗∗∗^*p* < 0.001.*

### Electrophysiological Data of the Go/Nogo Task

**FIGURE 1:**
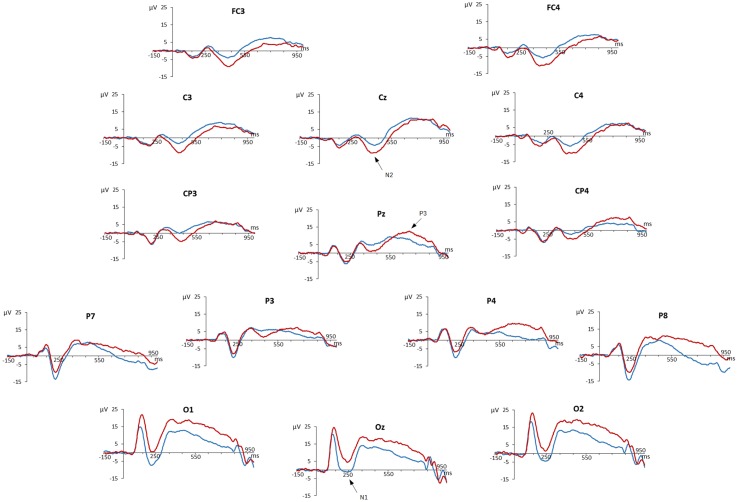
**Event-related potentials of the Go/Nogo task.** Amplitudes (in millivolt) of grand average stimulus-locked waveforms are shown for all analyzed 15 electrode sites. Waveforms of ‘go’ trials are blue-colored, those of ‘nogo’ trials are red-colored.

Grand-averaged ERPs at all analyzed 15 electrode sites are presented in **Figure 1.**

We observed a negative deflection at about 250 ms peaking most prominently at occipital sites, which was identified as N1 ERP component. ERPs of ‘go’ and ‘nogo’ trials started to diverge at occipital recording sites with more positive ERPs for ‘nogo’ trials. This Go/Nogo effect at occipital electrodes remained visible throughout the entire recording epoch.

Later, an N2 ERP component peaking at about 420 ms was present at fronto-central, but also at parietal electrodes, with a more pronounced negativity for ‘nogo’ than for ‘go’ trials. Finally, a P3/LPC peaking at about 700 ms was observed at parietal sites with more positive potentials for ‘nogo’ than for ‘go’ trials.

#### Go/Nogo Effects

Mean numbers of artifact-free EEG segments with correct responses, which were included in the analyses, were 59.85 (*SD* = 12.71) for the ‘go’ condition and 27.26 (*SD* = 6.33) for the ‘nogo’ condition.

For the N1 time window (184–284 ms) we found significant differences between ‘go’ and ‘nogo’ condition (Go/Nogo effects) as indicated by a main effect of condition at occipital [*F*(1,26) = 76.3, *p* < 0.001], parietal [*F*(1,26) = 10.5, *p* = 0.003] and midline [*F*(1,26) = 15.1, *p* < 0.001] electrodes. No significant Go/Nogo effects on the N1 were observed at fronto-central electrodes [*F*(1,26) = 1.5, *p* = 0.229]. Significant condition × electrodes interaction effects were observed at midline electrodes [*F*(2,52) = 47.2, *p* < 0.001]. In *post hoc* tests significant Go/Nogo effects were observed for Oz [*t*(26) = -8.1, *p* < 0.001] and Cz [*t*(26) = 2.5, *p* = 0.019], but not for Pz [*t*(26) = -0.9, *p* = 0.366]. Although the interaction condition × hemisphere was not significant, we tested whether the condition effect was reliable over either hemisphere. This analysis revealed significant condition effects for O1 [*t*(26) = -8.3, *p* < 0.001], O2 [*t*(26) = -7.6, *p* < 0.001], Par-left [*t*(26) = -3.3, *p* = 0.003] and Par-right [*t*(26) = -2.8, *p* = 0.009]. At occipital and parietal electrodes, the ‘nogo’ condition elicited a more positive potential than the ‘go’ condition, and at the Cz electrode, the ‘nogo’ condition elicited a more negative potential than the ‘go’ condition.

For the N2 time window (318–518 ms) significant Go/Nogo effects were observed at occipital [*F*(1,26) = 18.4, *p* < 0.001] and fronto-central electrodes [*F*(1,26) = 21.6, *p* < 0.001]. Significant condition × hemisphere interaction effects were observed at parietal electrodes [*F*(1,26) = 7.4, *p* = 0.012] and significant condition × electrodes interaction effects were found at midline electrodes [*F*(2,52) = 29.3, *p* < 0.001]. In *post hoc* tests significant Go/Nogo effects were observed for Oz [*t*(26) = -3.1, *p* = 0.005], Cz [*t*(26) = 4.6, *p* < 0.001] and Pz [*t*(26) = 2.1, *p* = 0.046], but not for the clusters Par-left [*t*(26) = 0.9, *p* = 0.398] or Par-right [*t*(26) = -1.8, *p* = 0.081]. Although the interaction condition × hemisphere was not significant, we tested whether the condition effect was reliable over either hemisphere. This analysis revealed significant condition effects for O1 [*t*(26) = -4.4, *p* < 0.001], O2 [*t*(26) = -3.9, *p* < 0.001], Fcentral-left [*F*(1,26) = 4.6, *p* < 0.001] and Fcentral-right [*F*(1,26) = 3.9, *p* < 0.001]. At occipital electrodes, the ‘nogo’ condition elicited a more positive potential than the ‘go’ condition. At lateral and midline fronto-central as well as at midline parietal electrodes, the ‘nogo’ condition elicited a more negative potential than the ‘go’ condition.

For the P3/LPC time window (600–800 ms) we observed significant Go/Nogo effects at occipital [*F*(1,26) = 35.1, *p* < 0.001], parietal [*F*(1,26) = 49.8, *p* < 0.001] and midline electrodes [*F*(1,26) = 8.0, *p* = 0.009]. Significant condition × hemisphere interaction effects were observed at parietal electrodes [*F*(1,26) = 15.2, *p* = 0.001]. At midline electrodes, the interaction between condition and electrode sites was significant [*F*(2,52) = 22.0, *p* < 0.001]. In *post hoc* tests significant Go/Nogo effects were observed for Oz [*t*(26) = -4.8, *p* < 0.001], Pz [*t*(26) = -2.8, *p* = 0.011], the clusters Par-left [*t*(26) = -3.4, *p* = 0.003] and Par-right [*t*(26) = -8.3, *p* < 0.001], but not for Cz [*t*(26) = 1.5, *p* = 0.160]. Although the interaction condition × hemisphere was not significant, we tested whether the condition effect was reliable over either hemisphere. This analysis revealed significant condition effects for O1 [*t*(26) = -6.0, *p* < 0.001] and O2 [*t*(26) = -5.5, *p* < 0.001]. At occipital and parietal electrodes, the ‘nogo’ condition elicited a more positive potential than the ‘go’ condition.

In the following correlation analyses only the electrodes/electrodes clusters with significant Go/Nogo effects were included.

#### Relation of ERP Go/Nogo Effects to Behavioral Executive Function Measures and Demographic Variables

The descriptive statistics for the Go/Nogo effects is shown in **Table 3.** **Table 3** also presents the correlations between the ERP Go/Nogo effects and the behavioral EF variables.

**Table 3:** Descriptive information for the ERP Go/Nogo effects and their association with behavioral EF measures (*N* = 27).

Ele. Pos.	*CE M*	*CE SD*	HTKS	DoG	Go/Nogo *d′*	Go Hits	Nogo FAs	Go RT	EF sum
**N1**								
O1	*–8.38*	*5.27*	–0.18 (–0.02)	–0.19 (–0.00)	–0.47^∗^ (–0.24)	–0.32 (–0.18)	0.44^∗^ (0.27)	–0.04 (–0.14)	–0.42^∗^ (–0.16)
O2	*–7.26*	*4.94*	–0.36 (–0.28)	–0.13 (–0.01)	–0.53^∗∗^ (–0.40^∗^)	–0.47^∗^ (–0.45^∗^)	0.44^∗^ (0.35)	0.40^∗^ (0.34)	–0.47^∗^ (–0.35)
Oz	*–7.21*	*4.63*	–0.18 (–0.13)	–0.11 (–0.02)	–0.48^∗^ (–0.45^∗^)	–0.37 (–0.43^∗^)	0.43^∗^ (0.36)	0.02 (0.00)	–0.40^∗^ (–0.31)
Par-left	*–2.53*	*3.95*	–0.12 (0.11)	–0.01 (0.37)	–0.41^∗^ (–0.09)	–0.23 (0.22)	0.31 (0.05)	0.07 (–0.05)	–0.26 (0.13)
Par-right	*–3.20*	*5.84*	–0.14 (–0.03)	0.02 (0.36)	–0.39^∗^ (–0.07)	–0.21 (0.06)	0.32 (0.04)	0.17 (0.20)	–0.26 (0.13)
Cz	*1.17*	*2.43*	–0.20 (0.05)	0.04 (0.12)	–0.16 (0.09)	–0.29 (–0.13)	0.11 (–0.05)	0.14 (0.13)	–0.13 (0.08)

**N2**								
O1	*–6.07*	*7.11*	–0.16 (–0.06)	–0.15 (–0.19)	–0.28 (–0.10)	–0.35 (–0.31)	0.16 (0.06)	–0.11 (–0.27)	–0.31 (–0.26)
O2	*–5.78*	*7.75*	–0.28 (–0.18)	–0.28 (–0.35)	–0.36 (–0.23)	–0.45^∗^ (–0.42^∗^)	0.19 (0.16)	0.07 (–0.16)	–0.44^∗^ (–0.43^∗^)
Oz	*–4.95*	*8.39*	–0.18 (–0.08)	–0.19 (–0.23)	–0.31 (–0.19)	–0.32 (–0.35)	0.22 (0.12)	–0.05 (–0.22)	–0.36 (–0.31)
Pz	*2.85*	*7.09*	–0.02 (0.02)	–0.13 (–0.02)	–0.20 (–0.12)	–0.06 (0.07)	0.23 (0.08)	–0.33 (–0.52^∗∗^)	–0.23 (–0.12)
Fcentral-left	*3.93*	*4.48*	0.06 (0.04)	–0.02 (0.05)	0.00 (0.04)	0.14 (0.20)	0.03 (–0.10)	–0.50^∗∗^ (–0.51^∗∗^)	–0.03 (0.06)
Fcentral-right	*3.49*	*4.65*	0.11 (–0.01)	0.08 (0.04)	0.06 (0.08)	0.29 (0.19)	0.07 (0.07)	–0.21 (–0.20)	0.04 (0.06)
Cz	*4.11*	*4.67*	–0.13 (–0.15)	–0.12 (–0.04)	–0.10 (–0.05)	0.09 (0.19)	0.20 (0.10)	–0.52^∗∗^ (–0.58^∗∗^)	–0.21 (–0.14)

**P3**								
O1	*–7.82*	*6.84*	0.03 (0.16)	–0.35 (–0.39^∗^)	–0.06 (–0.04)	–0.20 (–0.20)	0.01 (–0.03)	–0.09 (–0.18)	–0.16 (–0.17)
O2	*–8.12*	*7.74*	–0.12 (–0.04)	–0.46^∗^ (–0.47^∗^)	–0.02 (–0.03)	–0.25 (–0.37)	0.03 (–0.02)	–0.05 (–0.08)	–0.25 (–0.25)
Oz	*–6.60*	*7.19*	0.10 (0.08)	–0.35 (–0.39^∗^)	–0.06 (–0.15)	–0.11 (–0.35)	0.08 (0.12)	–0.22 (–0.24)	–0.16 (–0.27)
Par-left	*–3.77*	*5.84*	–0.29 (–0.30)	–0.33 (–0.29)	–0.05 (–0.12)	–0.21 (–0.15)	0.02 (0.04)	–0.21 (–0.19)	–0.25 (–0.32)
Par-right	*–8.74*	*5.50*	–0.30 (–0.18)	–0.39^∗^ (–0.30)	–0.30 (–0.19)	–0.47^∗^ (–0.46^∗^)	0.24 (0.17)	0.13 (0.03)	–0.42^∗^ (–0.29)
Pz	*–3.97*	*7.49*	–0.34 (–0.29)	–0.56^∗∗^ (–0.41^∗^)	–0.33 (–0.18)	–0.39^∗^ (–0.27)	0.26 (0.16)	–0.09 (–0.26)	–0.52^∗∗^ (–0.48^∗^)


*On the left mean values and standard deviations for the ERP Go/Nogo effects are presented. Right hand the Spearman’s rho correlation coefficients for the association between Go/Nogo effects and behavioral EF variables are shown. In brackets the partial correlation coefficients, controlling for children’s age and intelligence, are depicted.*

*Ele.Pos., electrode position; CE, Go/Nogo effect; HTKS, Head–Toes–Knees–Shoulders task; DoG, delay of gratification task; FAs, false alarms; RT, reaction times; EF sum, sum of z-standardized HTKS, DoG and ‘go/nogo’ *d′* scores; ^∗^*p* < 0.05; ^∗∗^*p* < 0.01.*

Greater occipital N1 Go/Nogo effects (i.e., greater differences between ‘go’ and ‘nogo’ N1) were significantly associated with higher response accuracy (i.e., higher *d′*, more hits, less false alarms) in the Go/Nogo paradigm. When we controlled for children’s age and intelligence, this association was reduced to insignificance for false alarms, but remained significant for *d′* and hits.

With regard to the N2 time window, more ‘go’ hits were significantly associated with a greater Go/Nogo effect at the right occipital site, when we controlled for children’s age and intelligence. No significant associations were observed for ‘nogo’ false alarms and ‘go/nogo’ *d′*. Faster ‘go’ reaction times were correlated with greater parietal N2 Go/Nogo effects when we controlled for children’s age and intelligence, and with greater fronto-central N2 Go/Nogo effects in both correlational analyses.

Higher DoG scores were significantly related to greater Go/Nogo effects in the P3/LPC time window at occipital and parietal electrodes in both correlational designs. Greater parietal P3/LPC Go/Nogo effects were also significantly associated with more ‘go’ hits in both correlational designs.

A higher EF sum score (i.e., sum of z-standardized HTKS, DoG and ‘go/nogo’ *d′* scores) significantly went along with greater occipital Go/Nogo effects in the N1 and N2 time window and with greater parietal P3/LPC Go/Nogo effects in both correlational designs.

We observed a significant association between the left parietal N1 Go/Nogo effect and children’s age (Par-left: ρ = -0.43, *p* < 0.05). Higher children’s intelligence (percentile rank of CPM) was significantly correlated with greater occipital Go/Nogo effects in the N2 time window (O1: ρ = -0.44, *p* < 0.05; O2: ρ = -0.42, *p* < 0.05).

### Emotional Availability

Descriptive statistics for the EA variables is shown in **Table 4.** *T*-scores ranged from 19 to 29, and EA CS ranged from 70 to 100. Adult structuring, adult nonintrusiveness and EA CS were significantly and positively correlated with children’s percentile rank of intelligence (**Table 4**). The EA CS and the EA scales except nonhostility were strongly positively intercorrelated (**Supplementary Table SI2**).

**Table 4:** Descriptive information for the emotional availability variables (*N* = 22).

Variable	*M*	*SD*	Observed range	CPM pr [ρ]
Adult sensitivity	26.59	2.17	21.00–29.00	0.38
Adult structuring	26.50	2.72	19.00–29.00	0.43^∗^
Adult non-intrusiveness	25.80	3.12	20.00–29.00	0.67^∗∗∗^
Adult non-hostility	28.05	1.81	23.00–29.00	–0.16
Child responsiveness	26.27	2.57	21.00–29.00	0.38
Child involvement	26.27	2.61	22.00–29.00	0.29
EA sum	159.48	11.35	133.00–174.00	0.38
EA CS	84.41	9.76	70.00–100.00	0.43^∗^


*Means, standard deviations, and observed ranges are depicted for the emotional availability (EA) variables (*T*-scores and EA CS). On the right Spearman’s rho correlation coefficients for the association between EA variables and the percentile rank of children’s intelligence are shown.*

*CPM, colored progressive matrices; M, mean; SD, standard deviation; pr, percentile rank; rho, Spearman’s rho correlation coefficient; ^∗^*p* < 0.05, ^∗∗^*p* < 0.01, ^∗∗∗^*p* < 0.001.*

#### Relation of Emotional Availability to Behavioral Executive Function Measures

We investigated the relations between EA variables and behavioral EF data. An overview of the results is presented in **Table 5.** Higher HTKS scores were significantly correlated with higher maternal nonintrusiveness and with a higher EA CS score in both correlational analyses. A higher EA CS score was also associated with a higher DoG score when we controlled for children’s age and intelligence and with a higher EF sum score in both correlational analyses. We found no significant associations between EA variables and response accuracy or reaction time in the Go/Nogo task.

**Table 5:** Association between emotional availability and behavioral EF measures (*N* = 22).

	HTKS	DoG	Go/Nogo *d′*	Go hits	Nogo FAs	Go RT	EF sum
Adult sensitivity	0.28 (0.28)	0.22 (0.37)	0.00 (–0.16)	0.16 (–0.14)	0.13 (0.06)	0.07 (0.09)	0.24 (0.22)
Adult structuring	0.24 (0.25)	–0.12 (–0.01)	0.17 (0.07)	0.13 (0.07)	–0.09 (–0.19)	0.06 (0.14)	0.17 (0.15)
Adult non-intrusiveness	0.51^∗^ (0.48^∗^)	0.04 (0.25)	0.19 (0.02)	0.36 (0.12)	0.06 (–0.12)	0.09 (0.38)	0.36 (0.39)
Adult non-hostility	0.16 (0.30)	0.17 (0.18)	0.06 (0.32)	0.12 (.27)	–0.04 (–0.23)	0.06 (–0.20)	0.21 (0.37)
Child responsiveness	0.17 (0.02)	–0.18 (–0.09)	0.08 (–0.05)	0.13 (–0.06)	0.15 (0.06)	0.16 (0.25)	0.08 (–0.05)
Child involvement	0.34 (0.16)	0.11 (0.21)	0.28 (0.27)	0.41 (0.22)	0.04 (–0.14)	–0.11 (–0.03)	0.33 (0.33)
EA sum	0.38 (0.27)	0.03 (0.11)	0.24 (0.10)	0.33 (0.13)	–0.01 (–0.07)	0.06 (0.07)	0.35 (0.26)
EA CS	0.43^∗^ (0.51^∗^)	0.27 (0.47^∗^)	0.20 (0.19)	0.32 (0.17)	–0.06 (–0.24)	0.04 (0.05)	0.43^∗^ (0.57^∗∗^)


*Spearman’s rho correlation coefficients for the association between emotional availability (*T*-scores and EA CS) and behavioral EF measures are shown. In parentheses the partial correlation coefficients are shown, controlling for children’s age and intelligence.*

*HTKS, Head–Toes–Knees–Shoulders task; DoG, delay of gratification task; FAs, false alarms; RT, reaction times; EF sum, sum of z-standardized HTKS, DoG and *d′* scores.*

*^∗^*p* < 0.05, ^∗∗^*p* < 0.01.*

#### Emotional Availability and ERP Go/Nogo Effects

The relations between EA variables and Go/Nogo ERP effects were examined (see **Table 6** and **Supplementary Table SI3**, for an overview of the results). Our data revealed several significant associations between EA variables and the N2 Go/Nogo effect, but no significant relations were found between EA and N1 or P3/LPC Go/Nogo effects.

**Table 6:** Association of emotional availability variables with N2 ERP Go/Nogo effects (*N* = 22).

Ele. Pos.	A-sensitivity	A-structuring	A-non-intrusiveness	A-non-hostility	C-responsiveness	C-involvement	EA sum	EA CS
**N2**								
O1	–0.48^∗^ (–0.25)	–0.54^∗∗^ (–0.47^∗^)	–0.59^∗∗^ (–0.50^∗^)	–0.21 (–0.30)	–0.50^∗^ (–0.39)	–0.25 (–0.13)	–0.54^∗∗^ (–0.45^∗^)	–0.44^∗^ (–0.18)
O2	–0.48^∗^ (–0.26)	–0.55^∗∗^ (–0.43^∗^)	–0.58^∗∗^ (–0.51^∗^)	–0.23 (–0.31)	–0.49^∗^ (–0.35)	–0.28 (–0.17)	–0.53^∗^ (–0.46^∗^)	–0.47^∗^ (–0.29)
Oz	–0.41 (–0.30)	–0.54^∗∗^ (–0.55^∗∗^)	–0.49^∗^ (–0.60^∗∗^)	–0.19 (–0.31)	–0.45^∗^ (–0.45^∗^)	–0.21 (–0.21)	–0.48^∗^ (–0.53^∗^)	–0.38 (–0.24)
Pz	–0.18 (–0.16)	–0.40 (–0.47^∗^)	–0.31 (–0.44^∗^)	0.03 (–0.08)	–0.37 (–0.46^∗^)	–0.02 (–0.09)	–0.31 (–0.34)	–0.17 (–0.09)
Fcentral-left	0.02 (0.02)	–0.19 (–0.23)	–0.05 (–0.20)	0.10 (0.13)	–0.25 (–0.32)	–0.18 (–0.19)	–0.19 (–0.12)	–0.10 (–0.05)
Fcentral-right	–0.08 (–0.19)	–0.36 (–0.46^∗^)	0.07 (–0.17)	0.28 (0.21)	–0.27 (–0.42)	–0.07 (–0.24)	–0.13 (–0.25)	–0.15 (–0.18)
Cz	–0.06 (–0.09)	–0.28 (–0.32)	–0.16 (–0.29)	0.18 (0.22)	–0.32 (–0.43^∗^)	–0.13 (–0.23)	–0.21 (–0.20)	–0.19 (–0.22)


*The Spearman’s rho correlation coefficients for the association between N2 Go/Nogo effects and emotional availability variables are shown. In parentheses the partial correlation coefficients, controlling for children’s age and intelligence, are presented.*

*Ele. Pos., electrode position; A, adult; C, child; ^∗^*p* < 0.05; ^∗∗^*p* < 0.01.*

In more detail, greater maternal sensitivity and a higher EA CS score went along with greater occipital Go/Nogo effects in the N2 time window. However, this effect did not hold, when we controlled for children’s age and intelligence. Higher maternal structuring and nonintrusiveness significantly correlated with greater occipital Go/Nogo effects in the N2 time window (in both correlational analyses). When we controlled for children’s age and intelligence, we additionally found that smaller midline parietal N2 Go/Nogo effects were significantly related to higher maternal structuring and nonintrusiveness, and that smaller right fronto-central N2 Go/Nogo effects were associated with higher maternal structuring.

Higher child responsiveness was significantly related to greater occipital Go/Nogo effects in the N2 time window in the zero-order correlation analysis, and when we controlled for children’s age and intelligence, higher child responsiveness was associated with greater midline occipital Go/Nogo effects in the N2 time window, and with smaller midline parietal and fronto-central N2 Go/Nogo effects.

A higher EA sum score was significantly associated with greater occipital Go/Nogo effects in the N2 time window, even when we controlled for children’s age and intelligence.

## Discussion

With this study, we aimed at investigating whether the quality of the mother–child interaction, assessed as EA, was associated with behavioral and electrophysiological measures of EFs in preschool children. We administered behavioral EF tasks, the Go/Nogo, the HTKS and the DoG task, to preschool children, and recorded ERPs during the Go/Nogo task, which taps response inhibition.

Children’s response accuracy in the Go/Nogo task highly correlated with HTKS performance, a measure of inhibition, shifting and working memory, and moderately correlated with DoG, a measure of impulse control. This result resembles the two factor structure of EFs in young children that was reported in Bernier et al. (2010, 2012). They found one factor loading on impulse control and one factor loading on working memory, shifting and inhibition, and a moderate correlation between these two factors. When we controlled for age and intelligence, the intercorrelations between the behavioral EF measures weakened. Additional data analyses (data not shown) indicated that the control for children’s age, but not for intelligence, accounted for this effect. HTKS and response accuracy scores were highly correlated with age. We think that in consequence of the small sample size only few children were of similar age, and this resulted in little variation in task performance over and above age. In contrast, impulse control, as measured by means of the DoG, was not significantly related to age. Previous research revealed that the ability to delay gratification shows moderate stability from 4 years of age to adolescence into adulthood four decades later (Mischel et al., 1989; Casey et al., 2011). Impulse control might represent a relatively stable personality trait from early childhood on.

We observed significant occipital, parietal, and fronto-central Go/Nogo effects in the N1 and N2 time windows. Further, we observed significant occipital and parietal Go/Nogo effects in the P3/LPC time window, in contrast to Jonkman et al. (2003), who reported absent ‘nogo’ P3 effects in children, aged 6–7 (Jonkman, 2006) resp. 9–10 (Jonkman et al., 2003). We additionally found a longer P3/LPC latency in our sample (600–800 ms) compared to Jonkman (2006) (reporting 440–480 ms) and Jonkman et al. (2003) (reporting 300–600 ms). In our sample the ERP components N1, N2, and P3/LPC showed longer latencies than usually observed in adults. This finding concords with developmental electrophysiological research that revealed decreasing latencies with age (Davis et al., 2003; Jonkman et al., 2003; Jonkman, 2006; Lamm et al., 2006); the decreasing latency with increasing age is probably paralleled by increasing myelination during the development of children’s cortices.

Furthermore, our results demonstrated that EA of the mother–child interaction is significantly associated with electrophysiological correlates of response inhibition during the Go/Nogo task in preschool children. However, our results yielded no significant associations between the behavioral response inhibition measures of the Go/Nogo task and the EA variables. Instead the EA variables maternal nonintrusiveness and EA CS were positively correlated with behavioral performance in the DoG and/or HTKS task.

Maternal structuring and maternal nonintrusiveness both refer to autonomy-fostering behaviors (Biringen and Easterbrooks, 2012b). High maternal structuring is characterized by adequate and consistent guidance and setting limits for the child, while high maternal nonintrusiveness encourages age-appropriate autonomy and simultaneously maintains an emotionally connected relationship. On the other hand, EFs are considered to be a crucial factor in human autonomy (Royall et al., 2002). It is an appealing notion, that parenting behavior which supports the child to practice autonomous actions, fosters EF proficiency. In support of this notion, previous developmental research depicted the importance of parental autonomy-support in the development of EF in children (Bernier et al., 2010, 2012; Bindman et al., 2015). Our data provide further evidence that maternal autonomy-support fosters EF skills in preschool children.

We found that children of higher autonomy-supportive mothers exhibited smaller fronto-central and parietal N2 Go/Nogo effects. The fronto-central N2 component is assumed to reflect higher-order inhibitory processes (Eimer, 1993; Kiefer et al., 1998) and/or conflict monitoring (Nieuwenhuis et al., 2003; Donkers and van Boxtel, 2004). Smaller fronto-central N2 Go/Nogo effects in a response inhibition task were related to increasing age in children (Davis et al., 2003; Jonkman et al., 2003; Jonkman, 2006). As we controlled for children’s age and intelligence, our results cannot be attributed to age effects. In our task, smaller fronto-central and parietal N2 Go/Nogo effects were also correlated with slower ‘go’ reaction times, and slower ‘go’ reaction times were in turn associated with less ‘go’ hits in our sample. However, a smaller fronto-central ‘nogo’ N2 amplitude was shown to be an indicator of better cognitive control over and above age (Lamm et al., 2006). Lamm et al. (2006) observed in children and adolescents that smaller fronto-central ‘nogo’ N2 amplitudes were associated with better performance in behavioral EF tasks, including a response inhibition task. Additionally, in a study with adolescents a reduced ‘nogo’ N2 amplitude was supposed to be an indicative of increased efficiency of the EF system (Stroth et al., 2009). We therefore postulate that higher maternal autonomy-support is related to electrophysiological indices of more efficient inhibitory control in our task over and above effects of children’s age and intelligence.

The N2 component typically peaks at fronto-central sites extending to parietal sites in adults (Kiefer et al., 1998). In our sample, we additionally observed occipital Go/Nogo effects in the N2 time window, with inverted polarity. In adults, the source of the fronto-central N2 is probably located in the inferior frontal cortex (Kiefer et al., 1998). In children, the N2 component might be generated in orbitofrontal and cingulate cortex (Lamm et al., 2006). Immature functioning of fronto-parietal circuits in young children has been suggested by some researchers based on electrophysiological and neuroimaging study results (Bunge et al., 2002; Ciesielski et al., 2004). Ciesielski et al. (2004) reported more pronounced parietal than frontal ERPs in children, ages 6–12, during the performance in a Go/Nogo task. In the fMRI study of Bunge et al. (2002), 8- to 12-year-old children exhibited insignificant prefrontal activation in a Go/Nogo task, while adults most robustly activated right ventrolateral prefrontal cortex (when contrasting ‘nogo’ and neutral trials). However, in an fMRI study contrasting ‘nogo’ and ‘go’ condition, 7- to 12-year-old children activated the same locations in the bilateral prefrontal cortex compared to adults, but with an extended volume in the dorsal and lateral prefrontal cortices (Casey et al., 1997). Hence, research about Go/Nogo-related prefrontal cortex functioning in young children yielded conflicting results. It is possible that the observed frontal and parietal N2 ERP originated from the same sources in frontal (and cingulate) cortex. However, the occipital ERP effect in the N2 time window, which showed a reversed polarity compared with the parietal and fronto-central effects, was likely generated in a more posterior brain region. In our task right occipital Go/Nogo effects in the N2 time window were associated with response accuracy (i.e., ‘go’ hits), whereas fronto-central and midline parietal N2 Go/Nogo effects were related to ‘go’ reaction times. This divergent associations might hint to different generators. More ‘go’ hits went along with greater occipital Go/Nogo effects in the N2 time window predominantly in the right brain hemisphere. Based on neuroimaging studies, a right-lateralized ventral fronto-parietal network has been identified, that involves the temporoparietal junction and is activated by the detection of low-frequency stimuli, like the ‘nogo’ stimuli in our task, which reorient attention (Corbetta and Shulman, 2002). The detection of low-frequency stimuli co-activates the bilateral dorsal fronto-parietal network that involves posterior parietal cortices. This latter network is implicated in goal-oriented sustained attention to stimulus attributes and it is involved in the selection of appropriate hand-responses to stimuli. Its neural activity is modulated by behavioral relevance and can additionally involve occipital activations (Le et al., 1998; Corbetta and Shulman, 2002). The observed occipital Go/Nogo effects in the N2 time window might therefore be related to attentional processes, generated in posterior parietal areas. However, it is somewhat difficult to explain why these attentional modulations were only related to behavioral accuracy in ‘go’ trials, but not to that in ‘nogo’ trials as well. Possibly, the relation is more indirect: More accurate responding in ‘go’ trials can simultaneously increase the difficulty of withholding the response in ‘nogo’ trials, because there is an increased tendency to execute a motor response. Thus, higher accuracy in ‘go’ trials without increased false alarms in ‘nogo’ trials can already indicate better response inhibition as a consequence of a more efficient fronto-parietal attention network. With regard to the association between higher maternal autonomy-support and greater bilateral occipital Go/Nogo effects in the N2 time window, we therefore suppose that higher maternal autonomy-support promotes attentional mechanisms that are implicated in better response inhibition.

Additionally, we observed that child responsiveness was comparably related to the Go/Nogo effects in the N2 time window as was maternal autonomy-support, with higher responsive children showing smaller fronto-central and parietal, and greater occipital effects. Smaller fronto-central N2 Go/Nogo effects might be electrophysiological indices of more efficient inhibitory control (Lamm et al., 2006; Stroth et al., 2009), and greater occipital Go/Nogo effects in the N2 time window might reflect more effective attentional processes as discussed above. The EA scale ‘child responsiveness’ resembles the concepts of secure attachment and ‘attachment-exploration balance’ from attachment theory, and depicts the child’s expression of healthy EA (Biringen and Easterbrooks, 2012b). A behavioral study reported that child attachment security predicts EF task performance (with regard to inhibition, working memory and shifting) in 3-year-olds (Bernier et al., 2012). Our results reveal for the first time the electrophysiological correlates that might underlie this association, and they provide further support that secure child attachment fosters EF-related proficiency.

Maternal sensitivity and EA CS went along with greater occipital Go/Nogo effects in the N2 time window. Maternal sensitivity in the EA framework resembles the attachment operationalization of maternal sensitivity, but emphasizes the quality of emotional exchanges and the dyadic nature to a higher extent (Biringen and Easterbrooks, 2012b). The EA CS is defined as summary of EA, stressing maternal sensitivity and child responsiveness. The association of both EA variables with the Go/Nogo effects in the N2 time window vanished when we controlled for children’s age and intelligence. Additional data analyses (data not shown) indicated that children’s intelligence accounted for this effect. Interestingly, children’s intelligence moderately correlated with maternal sensitivity (insignificantly) and significantly with EA CS. Maternal structuring and nonintrusiveness showed comparable or even stronger, significant associations with children’s intelligence. Our data thereby suggest that higher emotionally dyadic and autonomy-supportive mother–child-interactions promote children’s general intelligence, and this conforms with the proposition by Harrist and Waugh (2002) that dyadic synchrony facilitates children’s cognitive growth. Our results further suggest that maternal sensitivity compared to maternal autonomy-support affects children’s task-related ERPs by cognitive mechanisms that are more closely related to general intelligence than to EFs. Nevertheless, these interpretations can only be considered tentative, particularly because inter-rater reliability for maternal sensitivity and EA CS was suboptimal.

Despite the significant associations between maternal autonomy-support and N2 Go/Nogo effects, we observed no comparable link to behavioral performance in the Go/Nogo task (neither for maternal autonomy-support nor for any other EA variable). Although autonomy-support was related to Go/Nogo effects in the N2 time window similarly to ‘go’ hits and to ‘go’ reaction times, there was no equivalent association with behavioral performance (i.e., more ‘go’ hits and slower ‘go’ reaction times). Two explanations for the missing association of maternal autonomy-support with behavioral measures of the Go/Nogo task are conceivable. One possible explanation would be that maternal autonomy-support fosters cognitive processes that annihilate each other with respect to behavioral performance. In fact, analyses of our behavioral data showed that more ‘go’ hits were moderately associated with faster, and not slower, reaction times in our sample. An alternative proposition would be that electrophysiological measures might reflect task-relevant cognitive processes with a higher sensitivity than behavioral outcomes (e.g., Stroth et al., 2009). Compensatory neural processes like higher activation (e.g., of fronto-central ‘nogo’ N2) or additional recruiting of other brain circuits could conceal behavioral consequences of suboptimal cognitive processing in children with less autonomy-supporting mothers. As a result, no behavioral differences would be observed depending on maternal autonomy-support. However, compensatory processes might break down if the cognitive load gets higher. The HTKS task can be regarded as a more complex and difficult task than the Go/Nogo task, because it involves shifting and working memory updating next to inhibition. We observed that children of less intrusive (i.e., more autonomy-supportive) mothers performed better in the HTKS task. This result could be interpreted in favor of our second explanation. It also dovetails with other research that demonstrated a positive association between maternal autonomy-support and performance in inhibition, shifting, and working memory tasks in 2- to 4-year-olds (Bernier et al., 2010, 2012; Bindman et al., 2015).

While the N2 Go/Nogo effects showed little association with response accuracy in our Go/Nogo task, occipital N1 Go/Nogo effects were significantly related to all response accuracy measures (i.e., ‘go/nogo’ *d′*, ‘go’ hits, ‘nogo’ false alarms). The N1 component reflects focused attention and is presumably implicated in response inhibition (Luck et al., 1990; Filipović et al., 2000; Lavric et al., 2004). In the fMRI study by Bunge et al. (2002) behavioral accuracy was related to occipital, parietal, and temporal activation in children, and in adults only to occipital activation. A meta-analysis of fMRI studies revealed that successful response inhibition in a Go/Nogo paradigm activates bilateral occipital regions, next to right superior medial wall (pre-SMA) and precuneus (Simmonds et al., 2008). According to Friedman et al. (2009), variance in inhibition proficiency is mostly explained by the common EF factor, which might in turn be synonymic with an executive attention system (Norman and Shallice, 1986; Engle and Kane, 2004; Posner and Rothbart, 2007). We observed that better behavioral performance in the Go/Nogo task went along with greater occipital N1 Go/Nogo effects, i.e., a larger N1 amplitude for ‘go’ trials compared to ‘nogo’ trials. As a higher N1 amplitude indicates higher focused attention (Luck et al., 1990), we postulate that increased focused attention in the early visual processing of the ‘go’ stimulus is a prominent indicator of better behavioral accuracy in children, including not only more ‘go’ hits, but also less ‘nogo’ false alarms. Our results thereby suggest that children’s focused attention plays a crucial role in successful response inhibition, because ‘go’ and ‘nogo’ stimuli were visually better differentiated. As inhibition is highly related to the common EF factor, our data are also in line with assumptions that attentional control might represent the common EF factor. Our results are also in line with findings that occipital activation (Bunge et al., 2002) and the N1 Go/Nogo effect (Filipović et al., 2000; Lavric et al., 2004) are important indicators of successful response inhibition in a Go/Nogo task. It should be noted that the occipital N1 Go/Nogo effects extended into the N2 and P3/LPC time windows as indicated by a slow positive potential shift of the ‘nogo’ amplitudes. To our knowledge, this occipital slow wave effect has not been reported before because previous work on Go/Nogo experiments focused on ERPs at parietal, central, and frontal electrodes. It is possible that this slow wave reflects sustained attentional processes underlying response inhibition.

N1 Go/Nogo effects were not related to EA variables, and the same applied to the P3/LPC Go/Nogo effects. The P3 Go/Nogo effect is assumed to be an indicative of response inhibition (Kiefer et al., 1998; Smith et al., 2008), and presumably related to motor inhibition and performance evaluation. We found that greater parietal and occipital Go/Nogo effects in the P3/LPC time window correlated with a higher DoG score. Our Go/Nogo task implicated response inhibition to an attractive ‘nogo’ stimulus (a cookie). Similarly, the DoG task demanded impulse control toward an appetizing stimulus. Our data suggest that individual differences in DoG performance are associated with variations in motor inhibition and performance evaluation. A greater parietal P3/LPC Go/Nogo effect was also correlated with more ‘go’ hits. In turn our behavioral data showed that more ‘go’ hits were associated with higher DoG scores. Our findings hence provide a link between behavioral as well as electrophysiological indices of better response accuracy and better performance in the DoG. With regard to the EA variables, we observed a moderately strong association between the dyadic quality of the mother–child-interaction (i.e., EA CS score) and the DoG score. Previous studies revealed that attachment security (Jacobsen et al., 1997), maternal responsiveness (Razza and Raymond, 2013), and autonomy-support (Razza and Raymond, 2013; Bindman et al., 2015) were associated with a higher ability to delay gratifications. Our results thereby partially support these previous findings. The ability to delay gratifications was originally considered as an indicator of impulse control, but recent research demonstrated that it is also an indicator of interpersonal trust (Michaelson et al., 2013). A secure child attachment as well as highly dyadic mother–child-interactions feed the child with experiences of predictable and trustworthy others, and this might increase the children’s proclivity to postpone a gratification.

### Limitations and Future Directions

Our sample included 4- to 6-year-old children, covering an age span, during which considerable growth in EFs is observed. As discussed above, the rather small sample size limited the detection of significant EF effects over and above age especially in tasks, in which performance was highly correlated with age. A replication study in a larger sample would be desirable.

Furthermore, we observed little variation in educational degree in our sample. Our sample comprised predominantly highly educated parents, restricting thereby the generalizability of our results.

Our study was cross-sectional in design, and it would be interesting to follow-up into adolescence how the subsequent development of electrophysiological EF measures is modulated by the interaction quality between parents and child. In addition, future studies could include distress (e.g., cortisol levels; see e.g., Blair et al., 2011) and genetic markers (Barnes et al., 2011) in the examination of the association between neural correlates of children’s EFs and the early social environment.

## Conclusion

The current study addressed – to our knowledge for the first time – whether the parent–child interaction quality is associated with electrophysiological correlates of EFs in preschool children. Maternal autonomy-support and child responsiveness were significantly related to Go/Nogo effects in the N2 time window indexing response inhibition, over and above effects of children’s age and general intelligence. Our findings suggest that among other factors the quality of caregiver–child interaction shapes the functionality of the brain systems underlying EFs already during childhood. The present study therefore highlights the importance of parent–child interactions as a possible target of intervention programs aiming at improving EFs in children at risk.

## Author Contributions

HS-H supported data acquisition and study management, analyzed and interpreted the data, and drafted the manuscript. AZ contributed to central parts of the study conception and design. She planned and coordinated the acquisition of the families as well as coordinated and managed data acquisition and performed data management. She also did the analysis of the mother–child-interactions and revised the manuscript. CM supported data acquisition and EEG data processing, and revised the manuscript. UZ, MK, and AK initiated and conceptualized the collaborative study, and revised the manuscript. MK furthermore contributed to data analyses and interpretation as well as to drafting the manuscript. All authors approved the final version and agreed to be accountable for all aspects of the work.

## Conflict of Interest Statement

The authors declare that the research was conducted in the absence of any commercial or financial relationships that could be construed as a potential conflict of interest.

## Footnotes

1 http://www.sdqinfo.com/a0.html.

2 http://www.r-project.org/.

3 http://software.dell.com/products/statistica/.

## Abbreviations

HTKS: Head–Toes–Knees–Shoulders Task
DoG: delay of gratification task
EF: executive function
EA: emotional availability
EA CS: EA clinical screener
ERP: event-related potentials
LPC: late positive complex
M: mean
