# Supplementary material for: Emotional Availability Modulates Electrophysiological Correlates of Executive Functions in Preschool Children
Source: Front Hum Neurosci. 2016 Jun 23;10:299. doi: 10.3389/fnhum.2016.00299 (PMC4917527; doi:10.3389/fnhum.2016.00299)
Supplement: Supplementary file 1 [file Data_Sheet_1.DOC]

**Supplementary information**

**Table SI1: Association of the behavioral executive function measures with children's intelligence and maternal education (N=27)**

| Variable | CPM pr  [rho] | maternal education [rbis] |
| --- | --- | --- |
| HTKS [scores] | 0.31 | -0.12 |
| DoG [sec] | -0.05 | 0.01 |
| Go/Nogo d' | 0.16 | 0.16 |
| Go Hits [rel.fr.] | 0.36 | 0.11 |
| Nogo false alarms [rel.fr.] | 0.00 | 0.10 |
| Go RT [msec] | -0.15 | -0.20 |
| EF sum [z scores] | 0.16 | 0.02 |
| CPM [scores] | - | -0.06 |
| CPM [pr] | - | -0.37 |

HTKS, Head-Toes-Knees-Shoulders task; DoG, delay of gratification task; FA, false alarms; EF sum, sum of z-standardized HTKS, DoG and 'go/nogo' d'; CPM, Coloured Progressive Matrices; rel.fr., relative frequency; pr, precentile rank; rho, Spearman's rho correlation coefficient; rbis, pointbiserial correlation.

**Table SI2: Intercorrelations of emotional availability variables (N=22).**

|  | 1 | 2 | 3 | 4 | 5 | 6 | 7 | 8 |
| --- | --- | --- | --- | --- | --- | --- | --- | --- |
| 1 A -Sensitivity | - |  |  |  |  |  |  |  |
| 2 A-Structuring | .73*** | - |  |  |  |  |  |  |
| 3 A-Nonintrusiveness | .62** | .67*** | - |  |  |  |  |  |
| 4 A-Nonhostility | .37 | .19 | .32 | - |  |  |  |  |
| 5 C-Responsiveness | .66*** | .80*** | .72*** | .21 | - |  |  |  |
| 6 C-Involvement | .61** | .60** | .54* | .25 | .72*** | - |  |  |
| 7 EA Sum | .79*** | .82*** | .81*** | .40 | .90*** | .81*** | - |  |
| 8 EA CS | .90*** | .65** | .54** | .22 | .61** | .63** | .73*** | - |

Spearman's rho correlation coefficients are shown for the T-scores of the emotional availability (EA) scales and for the EA CS.

A, Adult; C, Child; *p<0.05, ** p<0.01,*** p<0.001

**Table SI3: Association of emotional availability variables with ERP Go/Nogo effects (N=22).**

| Ele.  Pos. | A-Sensi-tivity | A- Struc-turing | A- Nonintru-siveness | A- Non-hostility | C-Respon-siveness | C-Involve-ment | EA sum | EA CS |
| --- | --- | --- | --- | --- | --- | --- | --- | --- |
| **N1** | | | | | | | | |
| O1 | .11  (.07) | -.06  (-.17) | .05  (-.06) | -.08  (-.13) | .07  (.01) | -.01  (-.03) | -.02  (-.00) | .09  (.01) |
| O2 | .04  (.05) | -.20  (-.30) | .02  (-.14) | -.24  (-.23) | -.02  (-.07) | -.13  (-.20) | -.14  (-.23) | -.06  (-.09) |
| Oz | .06  (.05) | -.18  (-.28) | -.10  (-.29) | -.22  (-.22) | -.04  (-.13) | -.09  (-.19) | -.17  (-.22) | -.01  (-.07) |
| Par-left | .26  (.18) | .11  (.11) | .16  (.28) | -.03  (-.07) | .11  (.11) | -.02  (.01) | .08  (.15) | .25  (.23) |
| Par-right | .33  (.20) | .04  (-.19) | .28  (-.01) | .12  (.03) | .08  (-.16) | .03  (-.10) | 0.12  (-.09) | .22  (.15) |
| Cz | -.17  (-.05) | -.41  (-.42) | -.39  (-.28) | -.31  (-.42) | -.27  (-.20) | -.28  (-.10) | -.37  (-.32) | -.03  (.17) |
| **N2** | | | | | | | | |
| O1 | -.48*  (-.25) | -.54**  (-.47*) | -.59**  (-.50*) | -.21  (-.30) | -.50*  (-.39) | -.25  (-.13) | -.54**  (-.45*) | -.44*  (-.18) |
| O2 | -.48*  (-.26) | -.55**  (-.43*) | -.58**  (-.51*) | -.23  (-.31) | -.49*  (-.35) | -.28  (-.17) | -.53*  (-.46*) | -.47*  (-.29) |
| Oz | -.41  (-.30) | -.54**  (-.55**) | -.49*  (-.60**) | -.19  (-.31) | -.45*  (-.45*) | -.21  (-.21) | -.48*  (-.53*) | -.38  (-.24) |
| Pz | -.18  (-.16) | -.40  (-.47*) | -.31  (-.44*) | .03  (-.08) | -.37  (-.46*) | -.02  (-.09) | -.31  (-.34) | -.17  (-.09) |
| Fcentral-left | .02  (.02) | -.19  (-.23) | -.05  (-.20) | .10  (.13) | -.25  (-.32) | -.18  (-.19) | -.19  (-.12) | -.10  (-.05) |
| Fcentral-right | -.08  (-.19) | -.36  (-.46*) | .07  (-.17) | .28  (.21) | -.27  (-.42) | -.07  (-.24) | -.13  (-.25) | -.15  (-.18) |
| Cz | -.06  (-.09) | -.28  (-.32) | -.16  (-.29) | .18  (.22) | -.32  (-.43*) | -.13  (-.23) | -.21  (-.20) | -.19  (-.22) |
| **P3** | | | | | | | | |
| O1 | -.25  (-.10) | -.15  (-.10) | .00  (-.01) | .00  (.04) | -.12  (-.10) | -.11  (-.12) | -.10  (-.10) | -.22  (-.11) |
| O2 | -.21  (-.07) | -.15  (-.14) | -.09  (-.17) | -.02  (.02) | -.14  (-.12) | -.08  (-.11) | -.12  (-.15) | -.26  (-.15) |
| Oz | -.22  (-.09) | -.29  (-.28) | -.07  (-.30) | -.10  (-.05) | -.25  (-.28) | -.06  (-.08) | -.19  (-.29) | -.20  (-.09) |
| Par-left | -.15  (-.07) | -.09  (-.10) | -.11  (-.04) | -.01  (-.07) | -.17  (-.16) | -.14  (-.18) | -.19  (-.15) | -.25  (-.20) |
| Par-right | -.21  (-.06) | -.29  (-.29) | -.17  (-.18) | .01  (-.05) | -.23  (-.23) | -.29  (-.30) | -.28  (-.21) | -.35  (-.19) |
| Pz | -.01  (-.06) | -.06  (-.20) | -.21  (-.40) | -.05  (-.12) | -.17  (-.27) | -.09  (-.16) | -.17  (-.28) | -.11  (-.11) |

Spearman's rho correlation coefficients for the association between Go/Nogo effects and emotional availability variables are shown. In parentheses the partial correlation coefficients, controlling for children's age and intelligence, are presented.

Ele. Pos., electrode position; CE, Go/Nogo effect; HTKS, Head-Toes-Knees-Shoulders task; DoG, delay of gratification task; FAs, false alarms; RT, reaction times; EF sum, sum of z-standardized HTKS, DoG and d' scores;

* p<0.05; ** p< 0.01
